# Milieu Therapy in Patients with Dementia

**DOI:** 10.3390/jpm15060222

**Published:** 2025-05-29

**Authors:** Yong Tae Kwak, Young Soon Yang

**Affiliations:** 1 Department of Neurology, Hyoja Geriatric Hospital, Yongin 17089, Republic of Korea; 2 Department of Neurology, College of Medicine, Soonchunhyang University, Cheonan Hospital, Cheonan 31151, Republic of Korea; astro76@naver.com

**Keywords:** comprehensive geriatric care, dementia, long-term care, milieu therapy, therapeutic environment

## Abstract

This review explores the origins and principles of milieu therapy, which is traditionally rooted in psychiatric settings, and examines how these concepts can be adapted for patients with dementia. While milieu therapy initially focused on long-term psychiatric inpatients, the increasing prevalence of dementia—often with complex neuropsychiatric symptoms and extended care needs—warrants a renewed look at structured therapeutic environments. Unlike psychiatric conditions that may show significant improvement with medication, dementia typically involves progressive cognitive decline and multiple comorbidities, calling for a greater emphasis on safety, predictability, and emotional support. Core principles—therapeutic environment, social interaction, consistency, shared responsibility, empowerment, and individualized interventions—can be tailored to address dementia-specific needs, including orientation aids, simplified routines, and nonverbal communication strategies. Moreover, considering that dementia predominantly affects older adults, comprehensive geriatric care becomes crucial, requiring a collaborative team approach that includes medical, psychiatric, and rehabilitative expertise. In such environments, the focus shifts from cure to maximizing well-being, dignity, self-control, and residual abilities, underscoring the relevance of milieu therapy in modern dementia care.

## 1. Introduction

Dementia is one of the most pressing global health challenges, with an estimated 57 million people currently affected worldwide and nearly 10 million new cases each year. According to the World Health Organization, this number is projected to reach 139 million by 2050, largely due to global population aging (WHO, 2021) [1]. These statistics underscore the urgent need for effective, patient-centered care strategies such as milieu therapy. Most patients with dementia, including those with Alzheimer’s disease, display various types of behavioral and psychological symptoms as their illness progresses. Eventually, due to these symptoms, caring for them at home becomes difficult, and they often need to be transferred to long-term care facilities such as nursing homes or nursing hospitals for treatment [2,3,4]. In the West, such long-term care and treatment facilities have existed for psychiatric patients since the 1700s in the form of asylums [5], and studies on relationships with patients who have behavioral and psychological disturbances have been conducted in these contexts. Particularly up until the mid-20th century, the importance of environment and context in understanding and treating mental disorders was greatly emphasized. Therefore, treatments that encompassed all aspects of the patient’s environment were considered crucial in psychiatric care, and this approach was referred to as milieu therapy. The reasons for the development of milieu therapy are as follows. First, until the early 20th century, psychoanalytic and individual psychology schools—such as those of Freud, Jung, and Adler—and humanistic psychologists like Rogers attributed mental illness to environmental factors, suggesting that improving people’s social relationships and surroundings contributes to a supportive therapeutic setting. Based on this theoretical background, efforts to create a more humane and structured environment within psychiatric hospitals were active during the 1950s and 1960s, aiming to maximize therapeutic effects [6,7,8,9]. Second, in the past, there was a lack of effective psychiatric medications, so milieu therapy—a non-pharmacological intervention—became an important treatment method. Before effective antipsychotics emerged, longer hospital stays were common, making the therapeutic setting a central treatment component. Third, in the 1960s and 1970s, the therapeutic community movement began and spread. Rather than simply isolating or confining patients, this approach encouraged patients to actively participate in group therapy to regain social functioning. Within this trend, milieu therapy’s goal expanded beyond mere symptom control, aiming instead for patients to heal on their own within a therapeutic environment.

However, in modern times, the importance of milieu therapy seems to have decreased compared to the past. This is because effective medications—such as antipsychotics, antidepressants, and anxiolytics—have been developed for psychiatric patients, providing quick and reliable treatment methods. Moreover, modern psychiatric hospitals tend to focus on short-term admissions rather than long-term stays, making milieu therapy—more suitable for extended hospitalization—less central, as short-term symptom management and reintegration into society became the priority. Lastly, contemporary medicine emphasizes evidence-based therapy, and the difficulty in objectively measuring the effects of milieu therapy has also been a contributing factor.

Despite this, as the average lifespan has increased in recent years, the number of dementia patients has risen sharply, leading to more patients exhibiting behavioral disturbances or requiring help with daily living. Consequently, more individuals are being admitted to nursing hospitals or long-term care facilities, and the concept of milieu therapy, once applied to psychiatric patients, has become even more crucial in dementia care. Currently, many non-psychiatric physicians who primarily treat dementia patients in geriatric hospitals admit patients with diverse neuropsychiatric symptoms to specialized wards but often lack a clear concept of how the ward environment, beyond each patient’s individual problems, should be structured—or they hold differing views. Given the unique characteristics of dementia patients, physicians operating such wards or hospitals need to understand the concept of milieu therapy and apply it in practice. Even for psychiatrists, milieu therapy in dementia may be unfamiliar because, although dementia shares some features with psychiatric conditions, there are also fundamental differences. This review article aims to outline the general concept of milieu therapy and propose how it can be applied to dementia patients. Specifically, we will summarize the general concept of milieu therapy and suggest ways to integrate it into the care of patients with dementia.

### 1.1. What Is Milieu Therapy?

Milieu therapy is a structured treatment approach primarily used in psychiatric inpatient wards or residential treatment facilities. It involves deliberately designing the entire social, cultural, and physical environment—including patient living spaces, staff interactions, and peer relationships—to support the therapeutic process. The term “milieu”, derived from French and meaning “middle” or “surroundings”, emphasizes the significance of the treatment setting itself as an integral part of care, rather than relying solely on pharmacotherapy or specific clinical interventions [10,11]. Although milieu therapy emphasizes structured group environments, it also incorporates individualized interactions and care strategies tailored to each patient’s psychological and functional needs.

### 1.2. Historical Background

The origins of milieu date back to the 1700s. Dr. Philippe Pinel observed a reduction in patient violence when they were allowed free movement in a psychiatric hospital in Paris, highlighting the influence of the hospital’s structural environment on patient behavior [5]. It is also worth noting that similar values were embedded in traditional non-Western settings. For example, in pre-industrial Korean village communities, people with behavioral or psychological difficulties were often cared for within the family or local community, guided by Confucian ideals of social harmony and collective responsibility. Even in the absence of formal psychiatric frameworks, these communities fostered emotional containment and meaningful roles for individuals, reflecting elements now recognized in therapeutic milieus. During the late 19th and early 20th centuries, with the rise of psychoanalytic treatment under Freud’s school, psychiatric hospitals evolved from mere places of physical treatment into spaces for exploring patients’ psychological conflicts and facilitating healing through psychotherapy. However, at that time, psychiatric hospitals still operated with some controlling practices within their social and institutional context, leading to patients experiencing psychological regression in such environments. Sullivan (Harry Stack Sullivan) and Menninger (Karl Menninger) noted that psychiatric symptoms decreased when patients interacted with certain people, emphasizing the importance of the therapeutic environment and interpersonal relationships [12,13].

Milieu therapy gained broader recognition in 1953 with Maxwell Jones’s concept of social therapy, forming the basis for the model of the therapeutic community [14]. Jones argued that the therapeutic environment is not merely a backdrop but an essential component of the treatment process. This therapeutic community flattened hierarchical structures in hospitals, fostering a democratic culture where patients could learn interpersonal skills and participate in decision-making. In the late 1970s, Gunderson proposed five key elements for creating an effective therapeutic environment: containment, support, structure, involvement, and validation [15].

One issue is that different scholars define milieu therapy in various ways, and its meaning can shift depending on the user’s interpretation. Therefore, to understand this theory, one must grasp three main concepts that shaped the development of milieu in the 20th century. First, influenced by Freud’s ideas in the early 20th century, the focus in treating mental disorders was on analytic and interpersonal therapy, which was implemented in the ward when patients were admitted. This narrower, classical meaning of milieu therapy emphasizes analytic and interpersonal approaches [6,7,8,9]. Second, Maxwell Jones’s idea of the therapeutic community evolved into the concept of “community-as-doctor”, seeing the hospital not merely as a place to care for patients but also as a space where they can actively participate in the treatment process and learn through social interaction, leading to transformation [14]. Therapeutic communities employed methods like flattening hierarchical structures, involving patients in decision-making, and group therapy, emphasizing social interaction and learning among patients for societal re-adaptation. Third, milieu as a sociological extension, termed the therapeutic milieu, explores how the hospital’s social and physical environment affects the treatment process [10]. The Ward Atmosphere Scale is one such tool that attempts to quantitatively assess how a ward’s therapeutic atmosphere influences patient outcomes, and numerous related studies have been conducted.

In summary, milieu can be categorized into (1) a narrower meaning of milieu therapy emphasizing analytic/interpersonal psychotherapy through individual patient–staff interactions; (2) a therapeutic community environment focusing on social learning and participation; and (3) a therapeutic milieu that highlights how the environment and atmosphere serve as integral parts of the treatment process (Table 1). These concepts are no longer strictly distinguished as they once were; rather, they have been absorbed into each other or adapted to specific conditions and settings, often receiving unique names. In this paper, these three concepts are treated collectively under the overarching term “milieu therapy” without strict differentiation.

### 1.3. Key Principles of Milieu

(1) Therapeutic Environment

All elements of the environment—spatial layout, activities, schedule, interactions between staff and patients—should be designed to support treatment goals. This therapeutic environment must be safe, structured, and predictable to reduce anxiety and build trust. Lighting, spatial layout, safety measures, and cultural aspects of the ward should all be considered carefully.

(2) Interaction Between Patients and Between Patient and Staff

Patients are encouraged to play an active role in communal living, such as attending group meetings or participating in communal decision-making and mutual support. Patients’ disease types, symptoms, and functional levels should be accounted for when assigning roles and responsibilities. For example, in a patient with schizophrenia who is stable, group activities can be used to practice communication skills or take on a leadership role, while another patient in need of more psychological support might engage in emotional expression or stress management activities. A patient with depression may strengthen positive emotions through peer interactions and experience a sense of achievement through daily tasks or cooperative activities. Such interactions allow patients to learn social skills, improve communication, and practice healthy coping mechanisms aligned with their treatment goals.

(3) Consistency and Routine

A key component is maintaining a consistent daily routine. Having a regular schedule provides patients with stability and helps reduce anxiety and confusion. Therefore, scheduled mealtimes, individual and group therapy sessions, leisure activities, and communal meetings should be systematically organized so that patients can live in a predictable environment.

(4) Shared Responsibility and Empowerment

Patients are not mere recipients of treatment; they should be encouraged to be active members of the community. This entails guiding patients to regulate their own behavior, respect others’ boundaries, and engage actively in problem-solving. Through this, their sense of personal responsibility and autonomy is enhanced, and their self-efficacy is strengthened.

(5) Individualized and Group-Based Interventions

Milieu uses both individual and group-based interventions, meeting each patient’s individual treatment needs while maximizing therapeutic benefits from interaction with peers. Patients learn to give and receive feedback, thereby developing self-awareness and interpersonal skills. For dementia in particular, individualized care is crucial. Since dementia entails a gradual loss of self-determination and control, respecting each patient’s unique preferences and habits during treatment helps maintain normalcy and dignity [16,17].

### 1.4. Are There Differences in Milieu for Dementia Patients Compared to Psychiatric Patients?

(1) Differences of the Causative Disease

The course of dementia varies depending on its type, but conditions such as Alzheimer’s disease, Lewy body dementia, and vascular dementia are generally chronic and progressive and often involve irreversible cognitive decline. Meanwhile, even in rapidly progressing dementias such as Creutzfeldt–Jakob disease (CJD), the application of milieu therapy principles—even for a short duration—may be necessary to help maintain the patient’s emotional stability and remaining abilities. By contrast, in mental disorders (e.g., schizophrenia, bipolar disorder, major depression, and anxiety disorders), symptoms often involve emotional dysregulation, abnormal thought patterns, mood disturbances, or maladaptive behaviors. Although these disorders may have biological origins, they do not typically feature the gradual cognitive decline seen in dementia. Many psychiatric patients can achieve stability or improvement of symptoms with appropriate medication, psychotherapy, and psychosocial support.

(2) Differences in Treatment Goals

These differences extend from the divergence in the nature of the diseases. In dementia care, the main goals are to maintain the patient’s quality of life, preserve residual cognitive and functional abilities, and provide comfort. Emphasis is placed on environmental modifications, regular routines, and supportive interactions to reduce confusion and enhance safety and well-being. On the other hand, in mental disorders, the goals are largely rehabilitative, focusing on alleviating psychiatric symptoms, developing coping strategies, improving social skills, and ultimately promoting independent living and symptom management for reintegration into the community. Given that most degenerative dementias progress continuously, the most critical factor for patients is their present well-being, highlighting the importance of a safe environment. While this concept aligns with the optimal healing environment that milieu aims for [18], it may be more important for a dementia patient to feel comfort, stability, and well-being rather than “healing” per se. Rather than seeing “healing” as the goal, it may be better understood as providing a therapeutic environment in which patients feel that they can still exert some control over their bodies and minds, thereby preserving their dignity, identity, and functional abilities for as long as possible. This involves creating a calm and familiar physical environment, offering meaningful activities suited to their cognitive level, and providing consistent emotional support. Essentially, it is not about reversing the disease’s progression but about maximizing the patient’s quality of life. The reasons why milieu is necessary for dementia patients can be summarized as follows: First, it provides a therapeutic community that addresses mental symptoms such as depression, anxiety, restlessness, and psychosis. Second, it minimizes regression resulting from cognitive decline and reduced physical function—such as decreased self-esteem and social withdrawal—to reduce the risk of an escalating cycle of neuropsychiatric symptom deterioration. Third, it offers constant, familiar stimulation in a stable setting to help maintain cognitive function and prevent exacerbation. Fourth, it provides ways for various community resources and families to actively engage in a therapeutic environment according to their circumstances, fostering better adaptation to hospital life and acceptance of symptoms and functional decline as part of life.

### 1.5. Milieu for Dementia Patients

Milieu originally developed to treat psychiatric patients who required extended hospitalization. However, as noted in the introduction, patients with dementia of an organic etiology such as Alzheimer’s disease often exhibit numerous neuropsychiatric disturbances similar to those in psychiatric disorders and may also require extended inpatient treatment. Accordingly, in some countries or regions, specialized wards for dementia patients are in operation, and in such settings, the core principles of milieu can also be applied to dementia care. For instance, recent research indicates that even if two wards share the same physical structure, the manner of operation can affect the patient’s length of stay and likelihood of returning home [19], suggesting that milieu can significantly impact dementia patients as well. Nonetheless, dementia patients differ from psychiatric patients in that they often have substantially impaired cognitive functions (e.g., memory, attention, judgment) and are frequently elderly, possibly with multiple comorbid conditions. Therefore, the therapeutic environment for dementia patients must be safer, simpler, and more predictable, and it must also meet their medical needs. Adapting the previously mentioned principles to better suit dementia might look like this (Table 2).

(1) Therapeutic Environment

For general psychiatric patients, the therapeutic environment must be safe and predictable, avoiding excessive stimuli to reduce anxiety and confusion. Since dementia patients have more pronounced cognitive deficits and impaired memory, with reduced orientation to time and place, additional considerations are needed in designing the environment. This includes keeping lighting bright and even, ensuring non-slip flooring, minimizing complex layouts to maximize safety, providing large-print signage or color-coding, using familiar photographs as visual cues, and adjusting sensory inputs to match the patient’s level so they do not become distressed by overstimulation or monotony. Above all, safety is the foremost consideration in creating a therapeutic environment for dementia patients.

(2) Peer and staff interaction

Psychiatric patients commonly practice social skills through communal activities or group meetings, improving coping strategies via interaction with peers and staff. However, dementia patients often struggle with complex communication due to impaired memory and attention, so group activities and interaction methods may need to be modified. Patients with mild dementia can engage in relatively simple discussions or games, while those with moderate to severe dementia may find sensory-focused activities (singing, painting, hand massage, etc.) more effective for nonverbal interaction. Several studies have shown that sensory stimulation can reduce agitation, improve mood, and enhance social engagement in dementia patients, particularly when tailored to individual preferences and cognitive levels [20,21]. Staff should use short, clear sentences and actively employ supportive facial expressions and gestures such as hand-holding to reassure and support the patient. If milieu therapy for psychiatric patients primarily focuses on restoring social functioning, milieu therapy for dementia centers on emotional stability and sensory-based experiences [22].

(3) Consistency and Routine

For psychiatric patients, having a set schedule and consistent daily rhythm is crucial for stability and anxiety reduction. This routine is even more important for dementia patients, encouraging them to eat and take medications at the same times each day, adhere to regular sleep and activity schedules, and thus minimize confusion. Schedules can be visualized with images or large text so that patients can check them independently, and any environmental changes or new activities should be introduced gradually on a small scale to preserve predictability and safety. Over time, dementia patients become more comfortable with the daily routine, which can reduce confusion-driven anxiety.

(4) Shared Responsibility and Empowerment

Psychiatric patients can enhance autonomy and responsibility by directly participating in communal decision-making. For instance, they might decide which group activities to undertake or help set rules. By recognizing problems and exploring solutions, they adopt a more proactive stance and gain confidence. However, dementia patients, due to memory loss and poor concentration, often cannot independently handle complex procedures or major responsibilities. Consequently, any empowerment strategy must be simplified, with immediate feedback. For example, assigning small tasks in daily life (e.g., placing utensils on the table before meals, watering plants) and providing immediate praise and positive feedback can instill a sense of accomplishment and belonging in the community. Offering binary choices—“Shall we wear the red hat or the blue hat today?”—minimizes confusion and fosters a sense of personal decision-making. Even with dementia, patients can maintain and strengthen residual skills and self-determination if they continue to fulfill roles within the community.

(5) Individualized and Group-Based Interventions

Psychiatric patients may receive interventions such as cognitive–behavioral therapy, art therapy, and group counseling to alleviate symptoms and develop social skills. However, because dementia patients have deteriorating cognitive and memory functions, interventions must be adapted to suit their specific needs. For instance, personalized activities based on past interests or remaining abilities—like music, art, or reminiscence therapy—can be effective. Additionally, simpler cooperative activities like rolling a ball, easy crafts, or singing can encourage interaction, allowing patients to support and encourage each other. This approach helps dementia patients maximize their remaining capabilities, fostering social bonds and emotional stability, and repeated feedback can reinforce positive behaviors.

(6) Comprehensive Geriatric Care

Finally, because most dementia patients are elderly, they are likely to have various internal and surgical comorbidities, in addition to neuropsychiatric symptoms. This necessitates not only milieu but also comprehensive geriatric care. In other words, many dementia patients have multiple medical conditions, most of which are degenerative and cannot be fully cured, continuing to progress. This often entails polypharmacy and increased risk of side effects, as well as limited life expectancy [23]. Therefore, treatment may also require medication management for assorted medical conditions, minor surgical interventions, and sometimes a hospice care approach. Hence, treatment teams must possess both psychiatric expertise and clinical acumen related to geriatric medicine.

## 2. Conclusions

Milieu therapy stemmed from the idea that the environment surrounding patients can itself yield therapeutic benefits in treating psychiatric disorders. As pharmacological treatments and various new therapeutic approaches emerged, milieu therapy has evolved and been modified according to different goals and circumstances rather than remaining in its original form. More recently, the principles of milieu have been applied to specialized areas such as substance and alcohol dependence, building concrete therapeutic environments, and dementia care similarly needs to evolve in specialized ways aligned with the disease’s characteristics. Nonetheless, core elements of milieu—creating a supportive environment, sustaining a predictable and stable daily routine, and fostering positive social relationships—remain effective, especially for dementia patients who experience cognitive decline and behavioral disturbances. Because dementia involves progressive cognitive impairment and limited life expectancy, milieu concepts, originally centered on psychiatric patients, should not be applied wholesale but instead tailored to address the physical, cognitive, and emotional needs of dementia patients. Although no randomized controlled trials or systematic reviews have directly evaluated milieu therapy in patients with dementia, related evidence exists. For example, studies on person-centered, non-pharmacological interventions demonstrate reductions in agitation and improvements in quality of life, supporting the clinical relevance of milieu principles in dementia care settings [24,25]. This requires adjusting and advancing traditional milieu approaches to suit dementia patients while incorporating comprehensive geriatric care, as well as various clinical and rehabilitative strategies, in an integrated manner. Ultimately, milieu can be a flexible approach for dementia patients, just as it has been adapted for alcohol and substance dependence. It can continue to develop by reflecting the unique needs of this patient population. This effort is crucial for improving patients’ quality of life, preserving their dignity, and reducing the burden on families and communities. Consequently, healthcare professionals should become sufficiently informed about milieu and contribute to its further development.

## Figures and Tables

**Table 1:** The classical concepts of milieu therapy, therapeutic milieu, and therapeutic community.

Category	Milieu Therapy	Therapeutic Community	Therapeutic Milieu
Definition	Utilizes the everyday hospital environment and interactions as a form of therapy	Provides treatment through communal participation of patients and staff	The overall atmosphere and environment of the ward serve as therapeutic factors
Focus	Individual interaction: Treating internal conflicts through direct patient–staff interaction	Social learning: Patients learn responsibility and autonomy through community participation and change	Environment and atmosphere: The structure and attitude of the ward facilitate patient recovery
Theoretical Roots	Psychoanalytic/Interpersonal theories (Menninger, Sullivan)	Sociological community theories (Jones, Main)	Research in sociology and environmental psychology (Greenblatt et al.)
Purpose	Understand and resolve patients’ psychological conflicts	Reintegration through community participation	Creation of a positive “therapeutic atmosphere”
Treatment Method	Staff immediately interpret and give feedback on patient behavior	Horizontal organization (reducing power differences between patients and staff)	Optimizing interaction between ward atmosphere and patients
	Formation of individualized therapeutic relationships	Emphasis on group decision-making and patient participation	
Staff Role	Build therapeutic relationships, observe, and analyze patient behavior	Leaders and cooperative participants in the community	Indirectly promote therapy through the ward’s atmosphere
Patient Role	Passive role in the treatment process	Active participant in therapy, experiences responsibility and decision-making	Respected individual whose positive attitude contributes to shaping a therapeutic atmosphere
Applicable Setting	Psychiatric wards, pediatric treatment wards	Therapeutic community centers, addiction rehabilitation facilities	More limited participation, mainly influenced by the environment
Commonalities	-The treatment environment is important.		
	-Interactions with patients are used therapeutically.		
	-Can be integrated with traditional treatments (medication, psychotherapy, etc.).		
	-Ward rules and atmosphere offer patients autonomy and responsibility.		
Differences	-Milieu Therapy: Emphasis on individual relationships and immediate therapeutic interventions.		
	-Therapeutic Community: Emphasis on group participation and social reintegration.		
	-Therapeutic Milieu: Emphasis on the harmony of the overall environment; staff use their interactions to foster a therapeutic atmosphere.		

**Table 2:** Comparison of elements of milieu therapy in psychiatric vs. dementia patients.

Milieu Therapy Element	Psychiatric Patients	Dementia Patients	Practical Recommendations for Dementia Care
Therapeutic Environment	Safe, predictable, minimal stimuli	Requires additional structural simplicity and enhanced orientation aids due to memory loss and disorientation (e.g., clear signage, familiar photos, even lighting)	Use non-slip flooring, clear signage, avoid overstimulation; prioritize safety and familiarity
Peer and Staff Interaction	Group discussions, skill-building through interaction	Modified approach due to cognitive decline; use short verbal cues, nonverbal methods, and sensory activities (e.g., music, touch)	Train staff in facial cues and gestures; tailor activities to dementia severity
Consistency and Routine	Regular schedule to reduce anxiety	Routines are even more crucial; visual schedules help compensate for memory deficits	Use image-based schedules, consistent meal/med times, gradual transitions
Shared Responsibility & Empowerment	Participation in group decision-making, responsibility for communal roles	Needs to be much simplified; offer binary choices and guided tasks with immediate feedback	Assign simple daily tasks; use praise to reinforce sense of belonging and autonomy
Individualized & Group Interventions	CBT, group therapy, expressive therapies	Activities must be tailored to remaining abilities; use reminiscence, music, simple group tasks	Choose activities based on past interests and remaining abilities
Comprehensive Geriatric Care	May or may not be needed depending on age/comorbidities	Essential due to advanced age, multiple chronic illnesses, and degenerative course	Incorporate interdisciplinary care; monitor medications; consider hospice and palliative needs if needed
